# Study on interaction mechanism of different atomic ratio of neodymium, arsenic and iron

**DOI:** 10.1038/s41598-021-83698-9

**Published:** 2021-02-19

**Authors:** Juncheng Mao, Run Huang, Chenghui Fu, Xiaodong Lv, Lihua He, Jinzhu Zhang

**Affiliations:** 1 School of Materials and Metallurgy, Guizhou University, Guiyang, 550025 People’s Republic of China; 2 Guizhou Province Key Laboratory of Metallurgical Engineering and Energy Process Saving, Guiyang, 550025 People’s Republic of China; 3 College of Materials Science and Engineering, Chongqing University, Shapingba District, No.174 Shazheng Street, Chongqin, 400044 People’s Republic of China

**Keywords:** Metals and alloys, Atomistic models

## Abstract

In this study, neodymium and arsenic were sealed into industrial pure iron cylinders at a temperature of 1223 K for 50 h. The interaction mechanism of the Nd–Fe–As system at various atomic ratios was investigated by optical microscopy, X-ray diffractometry, and scanning electron microscopy. Binary compounds Fe_12_As_5_, NdAs, Fe_2_As, and Fe_17_Nd_2_ were the main products formed, with traces of NdFeAs compounds. In addition, at high temperatures, As content affected the diffusion of Fe atoms; the diffusion of Fe increased with an increase in the atomic ratio. Furthermore, the diffusion ability of Nd was weaker than that of As. The major diffusion mechanism of Nd was through the Fe atomic vacancy mechanism. As mainly bind to Fe to form Fe and As compounds. The formation of ternary compounds was confirmed by laboratory experiments and mismatch calculations.

## Introduction

The source of residual arsenic in steel is mainly primary iron ore. Compared with iron, arsenic has a weaker oxidation potential, so it is difficult to remove arsenic from steel in the entire steelmaking process. China not only has a large amount of iron ore containing arsenic, but with the development of social economy, the scrap cycle of steel is gradually shortened. It is estimated that by 2050, the comprehensive utilization ratio of scrap steel will reach 80%^1^. Arsenic, as a deleterious element commonly occurring in steel, enriched at the grain boundary, which significantly deteriorate impact toughness, cold brittleness, and hot-working property of steel^2^. In actual production, high-quality iron ore or molten iron can be mixed^3^, based on the reduction theory, steel companies add calcium to remove arsenic from molten steel^4–8^. But the former is only an emergency solution, the latter is easy to form large spherical oxides, both production processes have drawbacks. Rare earth elements (including 15 lanthanide elements, scandium, and yttrium^9–11^ in the third subgroup of the periodic table) are widely used for the purification, metamorphosis, and alloying of metals^12,13^.The unsaturated outer electronic layer of rare earth elements exhibit unique structure and strong chemical activity, which can burst out a variety of electron energy levels and exhibit high “vitality” in its external performance. Due to their active chemical properties, unique electrical and magnetic properties, rare earth elements can react with As (with a low melting point) to form compounds with high melting point, thus improving the thermoplastic and mechanical properties of steel. Rare earth elements are regarded as a treasure of new materials.

According to the iron-neodymium (Fe-Nd) binary phase diagram^14^, the stable compounds that can be formed between Fe and Nd include Fe_17_Nd_2_ and Fe_2_Nd, and according to the Fe-As binary phase diagram, the stable compounds that can be formed between Fe and As include Fe_2_As, Fe_3_As_2_, FeAs, and FeAs_2_^15^, in a study on Nd and As compounds, the binary diagram^16^ shows that Nd_3_As, NdAs and NdAs_2_. Generally, the maximum solubility of As in Fe is approximately 10% at 1113 K^17^. However, the solubility decreases with a decrease in temperature and reduces to below 5% at room temperature^18–20^. For Re-Fe-As ternary system, the main products including REFe_4_As_12_, REFe_2_As_2_ (RE = La, Nd, Sm) have been widely reported. Different atomic ratios of cerium (lanthanum), Fe, and As form the ternary compound RE_12_Fe_57.5_As_41_ (RE = La, Ce) and FeAs at 1173 K^21,22^, whereas the Re-Fe-As ternary system forms La_10_Fe_50_As_40_^23^ at 1223 K. In recent years, one of products of RE-Fe-As ternary system named EuFe_2_As_2_^24–28^ has attracted significant attention. Xie^29^and Fu^30^ investigated the interaction of Nd–Fe–As system at high temperatures and found that the formation of the ternary compound NdFeAs depends on the formation of NdAs and FeAs_2_.

Therefore, in this study, the different atomic ratio of Nd:As were sealed in a cylinder block, which was specially processed using industrial pure Fe by melting, infiltration, and diffusion. The interaction between Nd, Fe, and As at high temperature and the mechanism for the generation of ternary compounds was investigated using metallographic microscope, scanning electron microscope (SEM), and X-ray diffraction (XRD). The generation of ternary compounds was partly confirmed by calculations and laboratory experiments.

## Materials and methods

**Figure 1:**
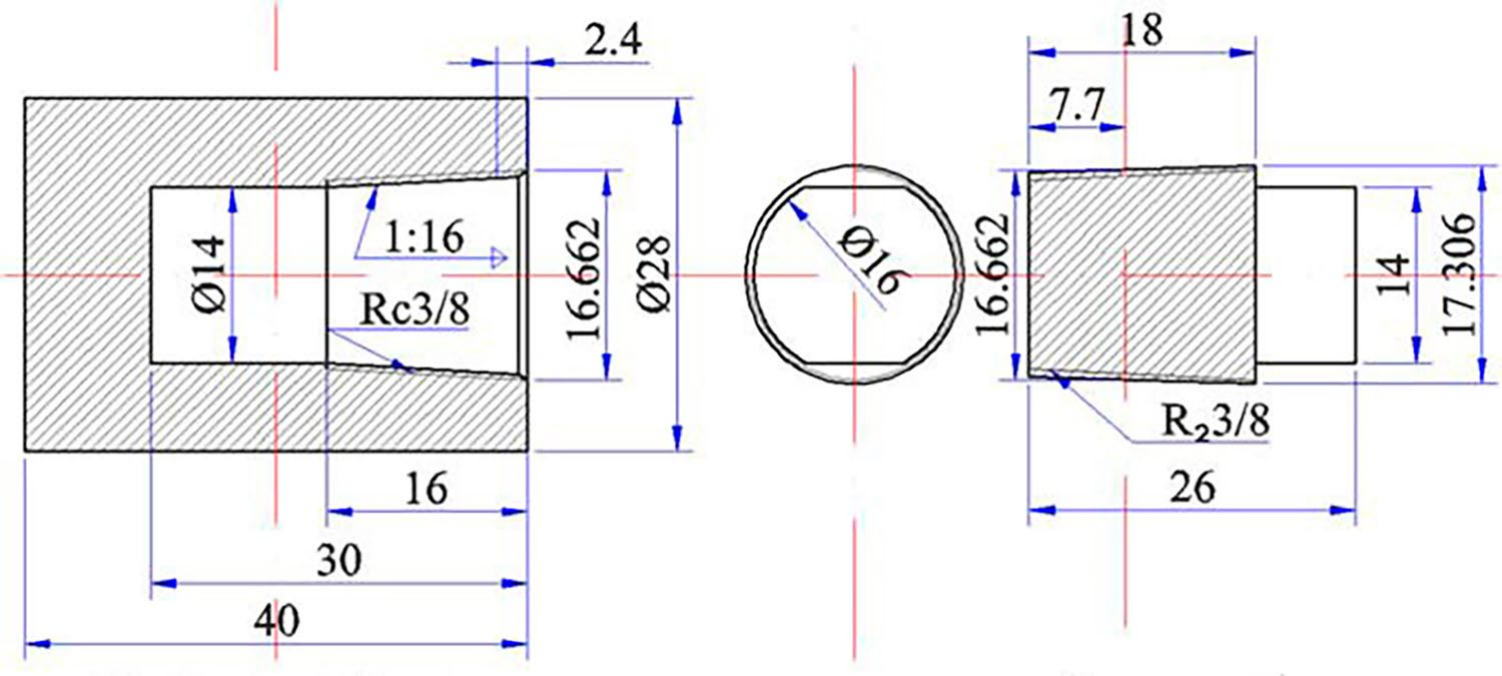
Schematic of the barrel-shaped cylinder and the screw plug.

Figure 1 shows the barrel-shaped cylinder composed of industrial pure Fe; its principal chemical composition (mass fraction) is as follows: 0.002% C, 0.02% Mn, 0.006% P, 0.004% S, 0.005% Al, and 99.95% Fe. Before the filling operation, the oxide layer on the Nd surface was removed, Nd metal block (purity > 99.9%) and As block (diameter < 1 mm, see Table 1) were filled into the industrial pure Fe cylinder block at various atomic ratios (1:1,1:2,1:3), the screw plug was welded by arc welding, and a high temperature sealant was applied to the weld to ensure it is properly sealed. Subsequently, The industrial pure Fe cylinder block was placed in a closed SRJK-2-9 tube resistance furnace and heated under high purity argon atmosphere. The experimental heating process is shown in Table 2, and it depended on the vapor pressure of As. After the heating process, the temperature of the furnace was reduced to room temperature (30 °C). Subsequently, argon flow into the furnace was stopped and the cylinder sample was taken out. Then, the outer side of the cylinder block was marked away from its bottom (at a distance of 16 mm); in the radial direction, it was sawed and divided into two parts, one of which was processed into metallographic samples and the other part was used for XRD analysis. The phase composition of the samples was analyzed using a PHILIPS X'-Pert PRO diffractometer, and the test parameters are as follows: Copper target, λ = 0.154056 nm, 40 kV operating voltage, 2°/min scanning speed.

**Table 1:** Masses of Nd and As and the atomic ratio of Nd:As for sample preparation.

Sample	Atomic ratio	Nd (g)	As (g)	T (K)	T (h)
1#	1:1	6.5814	3.4185	1223	50
2#	1:2	4.9014	5.0985	1223	50
3#	1:3	3.9089	6.0911	1223	50

**Table 2:** Experimental heating process.

Experimental heating process	
$$\begin{aligned} & {\text{Room}}\;{\text{temperature}}\mathop{\longrightarrow}\limits^{{{2}\;{\text{h}}}}{773}\;{\text{K}}\mathop{\longrightarrow}\limits^{{{10}{\text{K/10}}\;{\text{min}}}}{923}\;{\text{K}}\mathop{\longrightarrow}\limits^{{{10}\;{\text{K/20}}\;{\text{min}}}}{983}\;{\text{K}}\mathop{\longrightarrow}\limits^{{{10}\;{\text{K/30}}\;{\text{min}}}} \\ & {1023}\;{\text{K}}\mathop{\longrightarrow}\limits^{{{10}\;{\text{K/1}}\;{\text{h}}}}{1073}\;{\text{K}}\mathop{\longrightarrow}\limits^{{{10}\;{\text{K/2}}\;{\text{h}}}}{1123}\;{\text{K}}\mathop{\longrightarrow}\limits^{{{10}\;{\text{K/5}}\;{\text{h}}}}{1173}\;{\text{K}}\mathop{\longrightarrow}\limits^{{{10}\;{\text{K/6}}\;{\text{h}}}}{1223}\;{\text{K}}\;{(50}\;{\text{h)}} \\ \end{aligned}$$	

## Results

### Metallographic analysis

**Figure 2:**
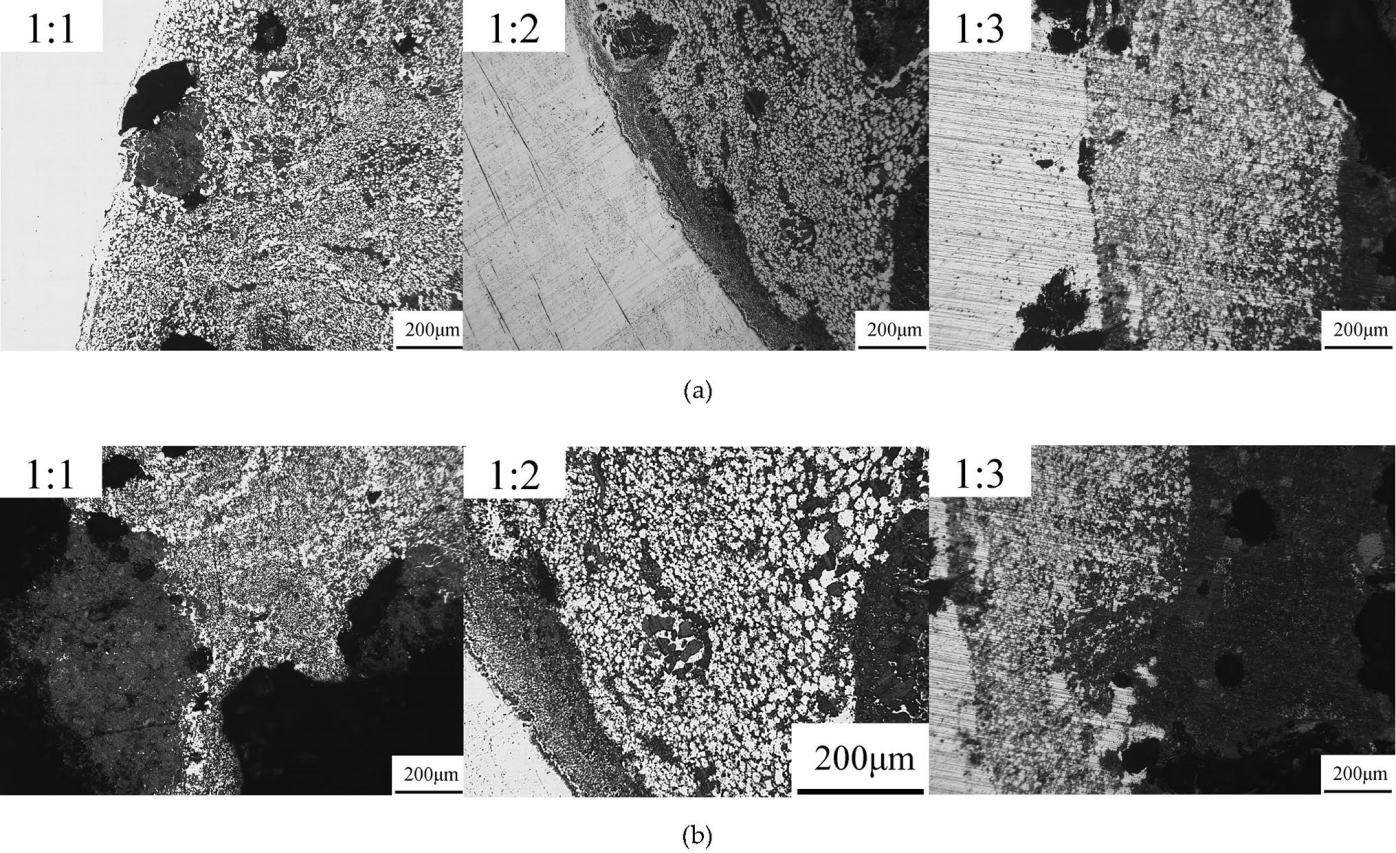
Metallographic pictures of atoms at different atomic ratio, (**a**) Pictures of the cylinder block’s neighboring area, (**b**) Pictures around its core area.

Figure 2 shows the metallographic images of the samples at various atomic ratios under an optical microscope. At a constant heating temperature and holding time, three types of contrast (grayish, gray, and black) were observed at different atomic ratios. The grayish part indicates the collective part of the cylinder, most of which was the industrial pure Fe, the gray part has a higher proportion of As, and the black part has a higher proportion of Nd. Figure 2a shows the neighboring area of the cylinder block, which indicates that the entire area was divided into two different parts: the cylinder matrix on the left and the core component on the right. In addition, inhomogeneous granular structures were formed at all atomic ratios (the right area), and they contained the same white liner as the cylinder matrix. With an increase in the atomic ratios, the symmetry of the entire granular structure increased and then decreased. In addition, with an increase in the diffusion of the grayish area, the diffusion moved farther away from the edge, which consequently reduced the diffusion. Particularly, with a decrease in the distance between the gray area and the edge of the boundary, the proportion of the gray area reduced. At high temperatures, the Fe atom diffused into the core area of the sample, while the Nd atom diffused into the matrix area, and the Fe and As atoms diffused together, and consequently, Fe atoms gradually formed a circular structure.

### Phase analysis

**Figure 3:**
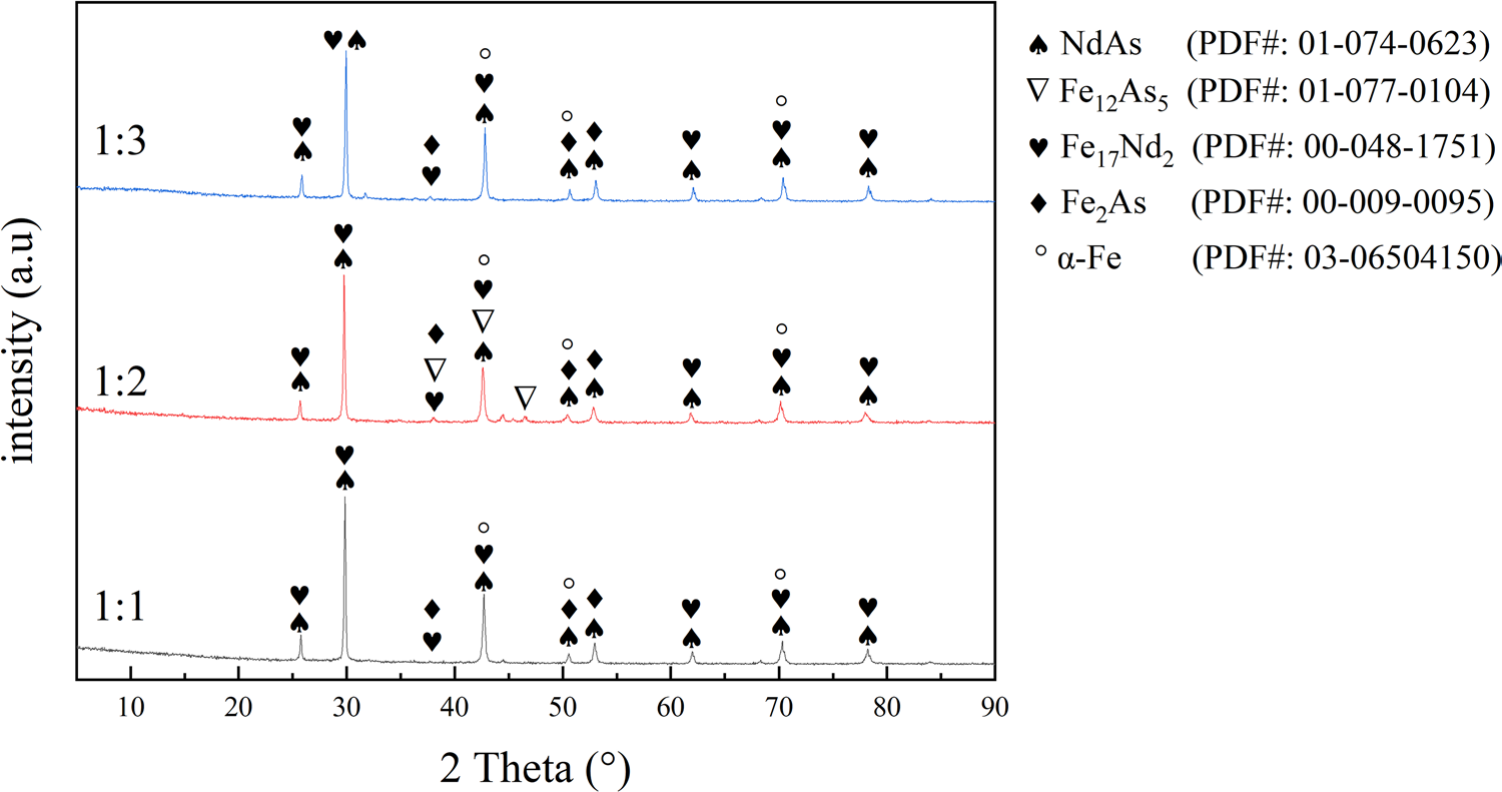
X-ray diffraction spectra of the samples at different atomic ratio.

For the phase analysis, the sample from the core area was ground in a mortar into a powder. Subsequently, the phase of the samples was characterized by XRD analysis, as shown in Fig. 3. The main diffraction peaks observed in the XRD spectra could be attributed to the formation of five types of compounds (Fe_12_As_5_, Fe_2_As, NdAs, Fe_17_Nd_2_, and α-Fe) in the ternary system of the high-temperature fusion samples. When the atomic ratio of Nd and As was 1:1 and 1:2, the intensity of the diffraction peaks was stable. During the experiment, As sublimed at high temperatures, while the highly reactive Nd reacted with As. Consequently, the number of compounds (NdAs) increased. As the experiment progressed, a small amount of As diffused into the external matrix of the cylinder block, and Fe diffused to its core area and reacted with As, forming As compounds (Fe_2_As).

XRD spectra of Nd–Fe–As powder compounds in the ternary system were not found in relevant literature. However, the energy dispersive spectroscopy (EDS) analysis suggests the formation of the ternary compounds (NdFeAs).

**Figure 4:**
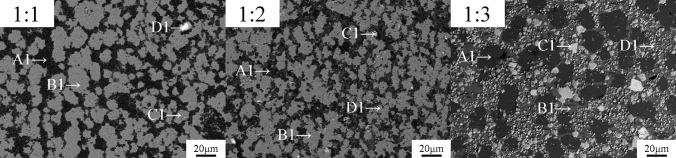
Backscattered electron maps of the sample at various atomic ratios (**a**) 1:1, (**b**) 1:2, (**c**) 1:3.

SEM analysis was carried out on the reduced sample, and EDS analysis was employed to analyze the image at different contrasts. As shown in Fig. 4, the samples are mainly distributed in four types of contrast phase (black, dark gray, light gray, and white), which are identified by A, B, C, and D, and each contrast phase tissue was investigated using EDS analysis; the results are shown in Table 3. The microstructure of the black, dark gray, and light gray contrast had an irregular shape. In addition, the gray and light gray components were mainly composed of Nd and As, combined with the XRD spectra of Nd and As, this result indicates that the contrast phase structure was a NdAs crystal structure, whereas the black contrast phase structure was mostly saturated As α-Fe solid solution.

**Table 3:** EDS analysis results of different atomic ratio samples (at/%).

Positions	Nd	As	Fe	Positions	Nd	As	Fe	Positions	Nd	As	Fe
A1	45.39	48.93	5.69	A1	44.32	43.34	12.34	A1	0.25	9.07	90.68
B1	35.14	42.24	22.63	B1	39.98	39.33	20.69	B1	39.39	42.34	18.27
C1	21.11	21.89	57.00	C1	20.79	27.75	51.46	C1	50.79	46.96	2.24
D1	2.27	11.21	86.52	D1	8.50	18.87	72.63	D1	0.74	8.55	90.72

As discussed above, the products of Nd and As at different atomic ratios (1:1, 1:2, 1:3) contained Fe_12_As_5_, Fe_2_As, NdAs, and Fe_17_Nd_2_ in all the ternary systems. However, the amount of different phases changes with an increase in the atomic ratios, and the various contrast phase structures show a symbiotic relationship.

### Diffusion analysis of samples

**Figure 5:**
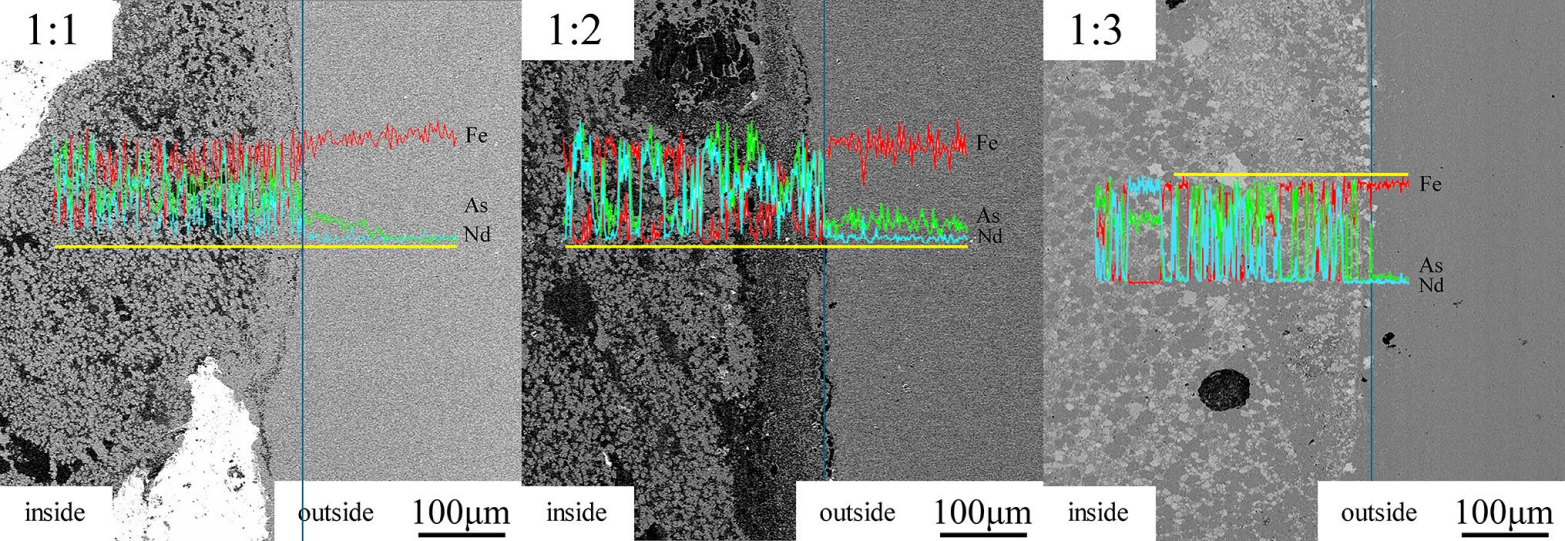
Sweep spectrum of the edge line of the cylinder samples at different atomic ratio.

Figure 5 shows the line-scanning atlas analysis of the transition areas of Nd and As at different atomic ratios. According to the graph, the three elements (Nd, Fe, As) were in a continuous distribution in a banded gradient. In addition, the amount of Nd in the white contrast region was significantly higher than that in other regions. Furthermore, the amount of Fe in the black contrast region was the highest, and As existed in all the contrast phases.

During the experiment, Fe diffused into the central region of the cylinder block, whereas the diffusion activation energy of As was lower than that of Fe^31^. In addition, As diffused via the formation of Fe and As compounds, and Nd diffused via its vacancy mechanism and the formation of the Fe and As compounds. Because the chemical potential of Nd increased with an increase in its content, and the solubility of As was maintained at 10% during experimental temperature, vacancies were produced during the sublimation process. The interaction between As and Nd produced NdAs compounds, and the remaining Nd and Fe formed Fe_17_Nd_2_ compounds to fill the vacancies. In addition, Fe vacancies were formed by thermal vibration; consequently, these vacancies provided a condition for the diffusion of Nd^32^. Furthermore, the grain size gradually decreased, thus increasing the grain boundary area. Simultaneously, the grain boundary vacancies increased accordingly. The combined effect of the generated vacancies, the Nd-generated solute-vacancy compounds, and the enhancement of the thermal diffusion rate leads to the segregation of Nd at the grain boundary.

### Planar mismatch calculation and analysis

According to the planar mismatch theory, the calculation result of the mismatch of two planes should be less than 6% to achieve good heterogeneous nucleation; heterogeneous nucleation is expected to occur if the mismatch is greater than 12%. The planar mismatch can be calculated using Eq. (1) ^33^.

1 $$\delta_{{\left( {hkl} \right)_{n} }}^{{\left( {hkl} \right)_{s} }} = \frac{1}{3}\mathop \sum \limits_{i = 1}^{3} \left[ {\frac{{\left| {d_{{\left[ {uvw} \right]_{s} }}^{i} \cos \theta - d_{{\left[ {uvw} \right]_{n} }}^{i} } \right|}}{{d_{{\left[ {uvw} \right]_{n} }}^{i} }}} \right] \times 100$$

where $\delta$ is the average of the three mismatches between the $\left( {hkl} \right)_{s}$ and $\left( {hkl} \right)_{n}$ planes; $d_{{\left[ {uvw} \right]_{s} }} \;{\text{and}}\;d_{{\left[ {uvw} \right]_{n} }} \;{\text{is}}\;{\text{the}}\;{\text{interatomic}}\;{\text{spacing}}\;{\text{along}}\;\left[ {uvw} \right]_{s}$, and $\left[ {uvw} \right]_{n}$ respectively; and *θ* is the angle between two corresponding direction.

**Figure 6:**
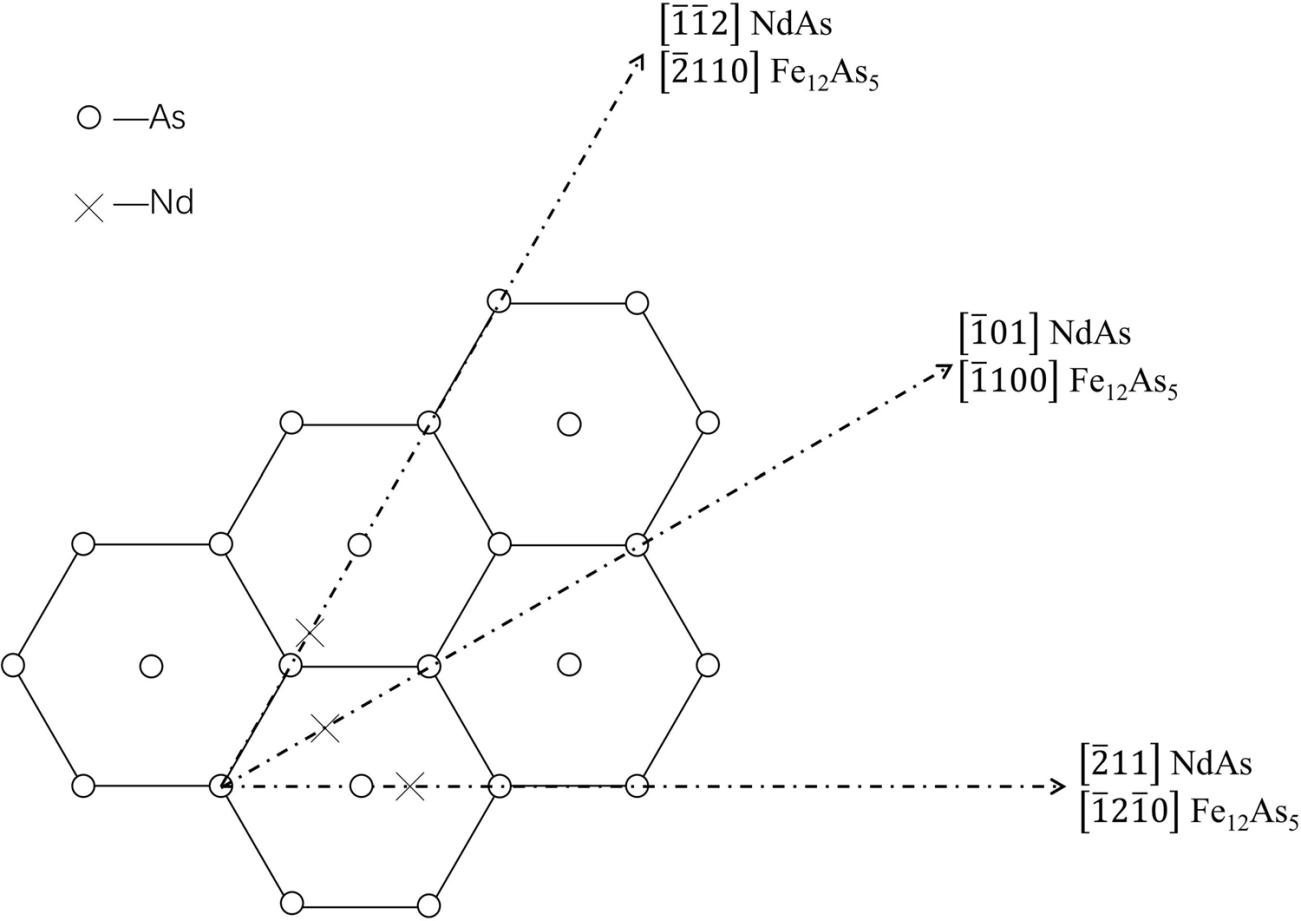
Crystallographic relationships of the Fe_12_As_5_ (0001) and NdAs (111) planes.

**Figure 7:**
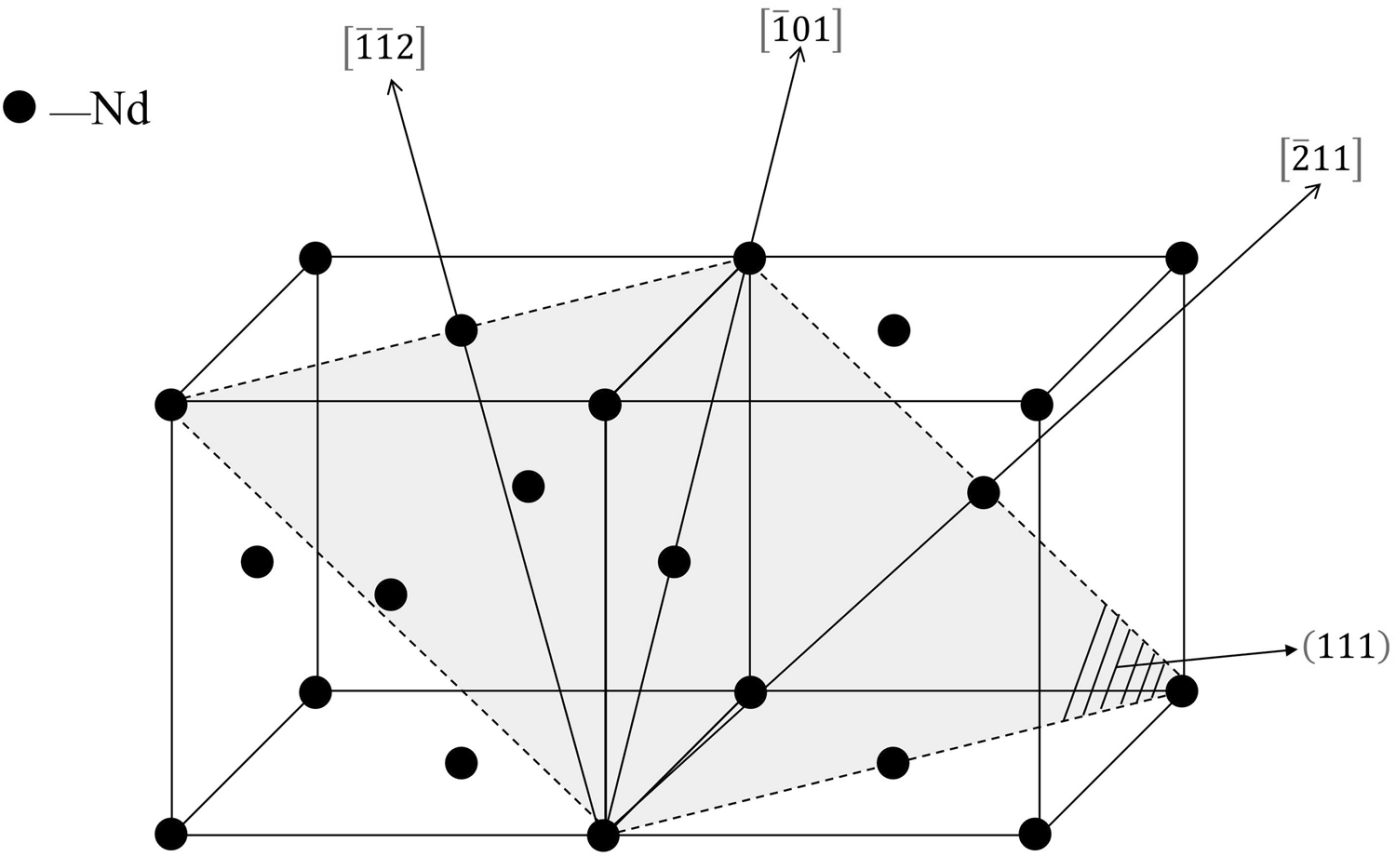
Diagram of plane and crystal direction of NdAs (111).

The atom matching diagram of the surface between the Fe_12_As_5_ (0001) and NdAs (111) planes is illustrated in Figs. 6 and 7. The former is indicated by “X” and the latter is indicated by “O”. The crystal parameters involved in the calculations are listed in Table 4^34^, and the specific calculation data are shown in Table 5. The calculation result of the two mismatched planes was 17.86%, indicating that Fe_12_As_5_ cannot be used as the effective heterogeneous nucleation core in the formation NdAs.

**Table 4:** Crystallographic parameters of possible compounds of Nd and As in steel.

Compounds	Crystal system	Lattice parameters (25 °C, nm)		
		$a_{0}$	$b_{0}$	$c_{0}$
Fe_12_As_5_	Hexagonal	0.6786	–	1.6301
NdAs	Cubic	0.5987	–	–
Fe_2_As	Tetragonal	0.3632	–	0.5981
Fe_17_Nd_2_	Hexagonal	0.8574	–	1.2464
NdFeAs	Tetragonal	0.39655	–	0.8575

**Table 5:** Calculation details of the lattice between Fe_12_As_5_ and NdAs compounds.

Interface	$\left( {0001} \right){\text{Fe}}_{12} {\text{As}}_{5} //\left( {111} \right){\text{NdAs}}$		
$\left( {hkl} \right)_{s}$	$\left[ {\overline{1}2\overline{1}0} \right]$	$\left[ {\overline{1}100} \right]$	$\left[ {\overline{2}110} \right]$
$\left( {hkl} \right)_{n}$	$\left[ {\overline{2}11} \right]$	$\left[ {\overline{1}01} \right]$	$\left[ {\overline{1}\overline{1}2} \right]$
$d\left[ {hkl} \right]_{s}$	0.6786	1.1745	0.6786
$d\left[ {hkl} \right]_{n}$	0.7331	0.4233	0.7331
*θ*	0	0	0
$d\left[ {hkl} \right]_{s} \cdot \cos \theta$	0.6786	1.1745	0.6786
δ (%)	64.11		

The calculation results of the mismatch are listed in the Table 6. The mismatch between Fe_2_As and NdFeAs was 8.33%, indicating the high probability for Fe_2_As to effectively act as the heterogeneous nucleation cores for the formation of ternary NdFeAs compound.

**Table 6:** Calculated planar lattice misfits among crystal faces of Nd–Fe–As inclusions.

Interface	δ (%)	Effectiveness
$\left( {0001} \right){\text{Fe}}_{12} {\text{As}}_{5} //\left( {111} \right){\text{NdAs}}$	64.11	Least effective
$\left( {0001} \right){\text{Fe}}_{12} {\text{As}}_{5} //\left( {0001} \right){\text{Fe}}_{17} {\text{Nd}}_{2}$	48.82	Least effective
$\left( {0001} \right){\text{Fe}}_{12} {\text{As}}_{5} //\left( {112.162} \right){\text{NdFeAs}}$	19.95	Least effective
$\left( {001} \right){\text{Nd}}$ As $//\left( {001.647} \right)$ Fe_2_As	22.96	Least effective
$\left( {111} \right){\text{Nd}}$ As $//\left( {0001} \right)$ Fe_17_Nd_2_	109.04	Least effective
$\left( {001} \right){\text{Nd}}$ As $//\left( {002.162} \right){\text{Nd}}$ FeAs	28.38	Least effective
$\left( {111.647} \right)$ Fe_2_As $//\left( {0001} \right)$ Fe_17_Nd_2_	37.21	Least effective
$\left( {001.647} \right)$ Fe_2_As $//\left( {002.162} \right)$ NdFeAs	8.33	Very effective
$\left( {0001} \right)$ Fe_17_Nd_2_ $//\left( {112.162} \right)$ NdFeAs	18.36	Least effective

## Conclusions

1. When the atomic ratios of Nd and arsenic were between 1:1 and 1:3 at a maximum temperature of 1223 K for 50 h, Fe_12_As_5_, Fe_2_As, NdAs, and Fe_17_Nd_2_ were formed. In addition, with an increase in the atomic ratios of Nd and As, the formation of the NdAs compounds decreased and the formation of Fe_2_As compounds increased (Fe_2_As was generated during the diffusion of As toward the cylinder block).
2. In the ternary system (Nd–Fe–As), the diffusion of Fe was dependent on the amount of As. In addition, the Fe atom diffused toward the core of the cylinder block, and its amount decreased with increase in the depth of its diffusion. Furthermore, with an increase in the proportion of Nd and As, the diffusion of As into the external matrix of the cylinder increased.
3. The EDS spectra suggest the formation of ternary compounds (NdFeAs), and the mismatch calculation indicated that the Fe_2_As can act as effective heterogeneous nucleation cores for the formation of ternary compounds (NdFeAs).
